# Whole-genome analysis of drug resistance and transmission patterns in *Mycobacterium tuberculosis* from Khyber Pakhtunkhwa, Pakistan

**DOI:** 10.1038/s41598-026-62482-7

**Published:** 2026-07-22

**Authors:** Muhammad Fayaz Khan, Jody Phelan, Anwar Sheed Anwar, Sadiq Noor Khan, Amjad Ali, Faryal Mehwish Awan, Ayesha Obaid, Dawood Ahmad, Afshan Saleem, Susana Campino, Taane G. Clark, Abdul Jabbar

**Affiliations:** 1 Department of Medical Lab Technology, University of Haripur, Haripur, Pakistan; 2 Department of Infection Biology, Faculty of Infectious and Tropical Diseases, London School of Hygiene & Tropical Medicine (LSHTM), Keppel St, London, WC1E 7HT UK; 3 Laboratory Hayatabad Medical Complex, Provincial Tuberculosis Reference, Peshawar, Pakistan; 4 Atta-Ur-Rahman School of Applied Biosciences, National University of Sciences and Technology, Islamabad, Pakistan; 5 Department of Microbiology, University of Haripur, Haripur, Pakistan; 6 Faculty of Epidemiology and Public Health, LSHTM, Keppel Street, London, WC1E 7HT UK

**Keywords:** *Mycobacterium tuberculosis*, Whole-genome sequencing, Drug resistance, Transmission, Pakistan, Bedaquiline, Computational biology and bioinformatics, Diseases, Genetics, Microbiology

## Abstract

The emergence of drug-resistant *Mycobacterium tuberculosis* strains presents a significant challenge to tuberculosis (TB) control programmes worldwide, particularly in high-burden countries, such as Pakistan. This study characterises the genomic diversity and drug resistance mutation profiles of *M. tuberculosis* isolates from Khyber Pakhtunkhwa using whole-genome sequencing (WGS). A total of 35 clinical *M. tuberculosis* isolates underwent WGS. Sequence data were quality-filtered and mapped to the H37Rv reference genome, characterising variants, including single-nucleotide polymorphisms (SNPs). Lineage classification, drug resistance profiling, and identification of resistance mutations were performed using TB-Profiler software. SNP-based clustering was used to infer recent transmission events. All four major *M. tuberculosis* lineages (L1–L4) were detected; however, more than half of the isolates belonged to lineage L3 (CAS, *n* = 19; 54.3%). Almost all isolates were drug-resistant (34/35), including pre-extensively drug-resistant (pre-XDR, *n* = 15; 42.9%) and multidrug-resistant (MDR, *n* = 14; 40.0%) strains. The most frequent resistance-associated mutations included *katG* S315T (27/35) and *inhA* c.-777C > T (4/35) for isoniazid resistance, *rpoB* S450L (24/35) for rifampicin resistance, *embB* M306I/V (18/35) for ethambutol resistance, and *gyrA* D94G (8/35) and A90V (5/35) for fluoroquinolone resistance. Compensatory mutations were observed in *rpoC* and *ahpC*. Mutations associated with bedaquiline and clofazimine resistance (*mmpR5* c.140dupA, c.138_139dupTG) were detected in single isolates. Interestingly, one isolate exhibited a deletion encompassing *mmpR5, mmpL5*, and *mmpS5*, potentially increasing bedaquiline susceptibility. Phylogenetic analysis using 6,635 genome-wide SNPs identified two transmission clusters with < 9 SNPs differences, suggesting ongoing local transmission. These results highlight the presence of drug-resistant *M. tuberculosis* strains in Khyber Pakhtunkhwa, encompassing both globally recognised and novel resistance-associated mutations. Our findings underscore the critical need to integrate WGS into TB surveillance and control programmes in Pakistan to guide targeted interventions and curb the spread of resistant strains.

**Supplementary Information:**

The online version contains supplementary material available at https://doi.org/10.1038/s41598-026-62482-7.

## Introduction

Tuberculosis (TB), a chronic infectious disease primarily caused by *Mycobacterium tuberculosis*, remains a major global health challenge. Despite advances in diagnosis, treatment, and prevention, TB continues to cause substantial morbidity and mortality, and drug-resistant forms increasingly threaten public health^1^. According to the World Health Organization (WHO) Global TB Report 2025, approximately 10.7 million people developed TB in 2024, leading to an estimated 1.23 million deaths^2^. Additionally, 4.5 million individuals remain undiagnosed or untreated, highlighting persistent gaps in TB control efforts. The emergence and spread of multidrug-resistant (MDR-TB) and extensively drug-resistant TB (XDR-TB)—affecting over half a million people globally—further complicate disease management. These forms require prolonged, toxic, and less effective treatment regimens, thereby hindering global TB elimination efforts^2^.

TB is a major public health concern in Pakistan, which ranks among the top countries for disease burden. The country accounts for approximately 5.7% of the global TB burden and is one of eight nations responsible for more than two-thirds of global TB cases, underscoring the urgent need for strengthened national control efforts^2^. Factors such as poverty, limited access to quality healthcare, high population density, low awareness, and the emergence of drug-resistant strains have perpetuated the TB epidemic^3^. Addressing these multifaceted challenges is crucial to reducing transmission and improving health outcomes, particularly in the highly affected province of Khyber Pakhtunkhwa. This region faces additional complexities due to its socio-political context, including tribal areas and a large refugee population, which present unique challenges to TB control. To effectively tackle both drug-susceptible and drug-resistant TB, the implementation of innovative diagnostic tools, improved treatment strategies, and strengthened public health programmes are essential^4^. Recent advances in molecular diagnostics, particularly whole-genome sequencing (WGS), have transformed our understanding of *M. tuberculosis* epidemiology and resistance mechanisms^5^. By elucidating the genetic diversity and transmission dynamics of circulating strains, WGS offers valuable insights to inform targeted interventions and enhance TB surveillance and control in Pakistan.

The *Mycobacterium tuberculosis* complex (MTBC) consists of ten distinct lineages (L1-L10), each with a unique geographic distribution. This phylogeographic pattern supports the hypothesis that these lineages have adapted to specific human populations^6^. For instance, lineage L2, characterised by its global dissemination, from Asia to Europe and Africa, is associated with increased transmissibility^7^. Conversely, L4, prevalent in Europe and southern Africa, correlates with regions with high TB and HIV co-infection rates^8^. While L3, encompassing Central Asian (CAS) strains, is predominantly found in South Asia^9^. Beyond geographic distribution, these lineages differ in their epidemiological and clinical characteristics^10^. Studies suggest potential differences in transmissibility and disease severity among lineages. Nevertheless, there is significant intra-lineage diversity, emphasising the complexity of TB epidemiology^10^. Distinct sets of genetic markers, including large sequence deletions (regions of difference) and single-nucleotide polymorphisms (SNPs), have been characterised to define and barcode the major lineages (L1–L10) as well as strain-specific sublineages^10^. These molecular signatures facilitate improved classification and enhance our understanding of strain-level variation. Recent advances in WGS have revolutionised the understanding of TB transmission dynamics. By identifying SNPs and other variants, researchers can construct phylogenetic trees and transmission networks, providing valuable insights into the spread of specific strains^11,12^.

Previous genomic studies in Pakistan have highlighted the predominance of *M. tuberculosis* lineage L3 strain types, providing clear evidence of ongoing transmission of MDR and (pre-)XDR isolates between 2003 and 2020 (n = 535)^13^. WGS analyses of isolates from this region have supported the transmission of drug-resistant strains^14^, revealing that rifampicin resistance often emerged before isoniazid resistance^15^. Furthermore, some pre-MDR isolates (resistant to either isoniazid or rifampicin, but not both) also carry fluoroquinolone resistance markers^15^. Subsequent studies based on collections before 2023 have consistently confirmed the circulation of MDR-TB and pre-XDR strains, with recurrent mutations conferring resistance to isoniazid (*katG* S315T; *inhA* − 15C > T, − 154G > A)^16^, rifampicin (*rpoB* S450L/W/F, R552, H445D/L/R, S874Y)^17^, pyrazinamide (*pncA*), and fluoroquinolones (*gyrA* D94G, A90V)^18^.

Building on this foundation, the current study analyses *M. tuberculosis* isolates collected in 2023 from Khyber Pakhtunkhwa using WGS. Our study characterises resistance mutations across 14 anti-TB drugs, elucidates transmission dynamics of drug-resistant strains within the region, and provides evidence to inform public health policies for TB control.

## Methods

### Study population, sample processing, and drug susceptibility testing

A total of 3859 sputum samples were collected between January and September 2023 at the Provincial TB Reference Laboratory, Hayatabad Medical Complex, Peshawar, Khyber Pakhtunkhwa, Pakistan. Patient data, including age, sex, and treatment regimen, were collected for each isolate. All clinical specimens were decontaminated using the standard N-acetyl-L-cysteine sodium hydroxide (NALC-NaOH) method within a Biosafety Level 3 (BSL-3) laboratory, adhering to GLI guidelines^19^. Subsequently, aliquots were inoculated on Lowenstein-Jensen (LJ) medium and in Mycobacterium Growth Indicator Tubes (MGIT). Positive cultures were confirmed using the Tbc ID system^20^. Additionally, acid-fast bacilli smear microscopy was conducted on sample aliquots using a Primostar-LED fluorescent microscope. Phenotypic drug susceptibility testing (pDST) was performed on all positive samples using the BD BACTEC MGIT 960 SIRE system with standardized drug concentrations; 1 µg/mL for rifampicin and 0.1 µg/mL for isoniazid, ethambutol (5.0 µl/ml), streptomycin (1.0 µl/ml), moxifloxacin (2.5 μg/ml), amikacin (1.0 μg/ml), kanamycin (2.5 μg/ml), capreomycin (2.5 μg/ml) and ofloxacin (2.0 μg/ml). Pyrazinamide susceptibility testing was performed using an established protocol^21^. A GeneXpert® system (Xpert MTB/RIF; Cepheid, USA) was used to confirm the identity of rifampin-resistant isolates.

### DNA extraction

Genomic DNA was extracted from *M. tuberculosis* isolate cultures, with 35 isolates selected by prioritising drug-resistant strains based on pDST results and DNA quality, assessed using spectrophotometry (NanoDrop) and fluorometry (Qubit). This convenience sample included the four major lineages (L1-L4). Bacterial colonies grown on LJ slants were used for DNA extraction following the standard CTAB-phenol–chloroform protocol. Briefly, bacterial cells were lysed using heat, enzymatic digestion, and chemical treatments. Proteins were removed by phenol–chloroform extraction, and DNA was precipitated with isopropanol. The purified DNA was subsequently washed with ethanol and resuspended in elution buffer^22^.

### Sequencing, QC and alignment

WGS of *M. tuberculosis* isolates was conducted using Illumina’s HiSeq platform (San Diego, CA, USA) at The Applied Genomics Centre, London School of Hygiene & Tropical Medicine, London, UK (genomics.lshtm.ac.uk). Sequencing quality was evaluated using FASTQC, and raw reads were trimmed with Trimmomatic^23^ (v0.30) to remove low-quality sequences. Subsequently, reads were mapped to the H37Rv reference genome (AL123456) using BWA mem^24^ (v0.7.19).

### Identification of lineages, resistance genes and associated mutations

Drug resistance profiles, associated mutations, and (sub)lineages were predicted in silico using TB-Profiler^25,26^ (v6.4.0), with support from a library of established mutations (https://github.com/jodyphelan/tbdb). The same TB-Profiler software was employed for in silico spoligotyping^27^. Mutation frequencies were compared with those in a global *M. tuberculosis* dataset (n = 152,408)^26,27^.

### Phylogenetic analysis

A whole-genome phylogenetic tree analysis of the 35 study strains was conducted to assess the genomic diversity of the sequenced strains. Consensus genomes were created using TB-Profiler and used as input to IQ-Tree^28^ (v3.0.1) with the GTR + G model employing pathogen mode and 1000 bootstrap replicates. Known non-unique regions of the genome (e.g. *PPE* genes) were excluded^29^. The tree was annotated and visualised using iTOL^30^.

### Ethical considerations

Ethical approval for this study was obtained from the Institutional Review Boards of The University of Haripur and the Provincial TB Reference Laboratory, Hayatabad Medical Complex, Peshawar, Pakistan. Informed consent was obtained from all study participants who provided sputum samples. All procedures adhered to the relevant guidelines and regulations. DNA samples were transferred under the conditions specified in a Material Transfer Agreement.

## Results

### Characteristics

Of the 3859 sputum samples analysed, 209 (5.4%) tested positive for *M. tuberculosis* using culture, MPT64 immunochromatographic testing, and fluorescent microscopy. Thirty-five isolates underwent WGS. The mean age of patients was 34.7 years, with 19 (54.3%) males. Most isolates originated from Peshawar (n = 16, 45.7%), followed by Dera Ismail Khan (n = 7, 20.0%), Abbottabad (n = 6, 17.1%), Mardan (n = 3, 8.6%), and Swat (n = 3, 8.6%) (Table 1).

**Table 1:** The characteristics of the sequenced *M. tuberculosis* isolates (n = 35).

Characteristic	N (median)	% (Range)
Patient age (years)	(35)	(23–57)
Patient male	19	54.3
*Region*		
Peshawar	16	45.7
Dera Ismail Khan	7	20.0
Abbottabad	6	17.1
Swat	3	8.6
Mardan	3	8.6
*Lineage*		
L3	19	54.3
L4	10	28.6
L2	5	14.3
L1	1	2.9
Genotypic [Phenotypic*] DR		
Isoniazid	32 [35]	91.4 [100]
Rifampicin	31 [30]	88.6 [85.7]
Ethambutol	27 [9]	77.1 [25.7]
Streptomycin	20	57.1
Fluoroquinolones	18	51.4
Pyrazinamide	20 [16]	57.1 [45.7]
Ethionamide	8	22.9
Bedaquiline	1	2.9
Clofazimine	1	2.9
Kanamycin	1	2.9
Pre-XDR-TB	15	42.9
MDR-TB	14	40.0
HR-TB	2	5.7
RR-TB	1	2.9
XDR-TB	1	2.9
Sensitive	2	5.7

DR, drug resistance; RR-TB, rifampicin-resistant TB; HR-TB, isoniazid-resistant TB; MDR-TB, multidrug-resistant TB; XDR-TB, extensively drug-resistant TB; * also, moxifloxacin (fluoroquinolone) 6 and amikacin (aminoglycoside) 4.

### Phenotypic resistance

Phenotypic drug susceptibility testing (pDST) showed that all isolates were resistant to isoniazid (n = 35, 100%), with additional resistance to rifampicin (n = 30, 85.7%), pyrazinamide (n = 16, 45.7%), ethambutol (n = 9, 25.7%), moxifloxacin (n = 6, 17.1%), and amikacin (n = 4, 11.4%) (Table 1).

### Genotypic profiles

**Fig. 1:**
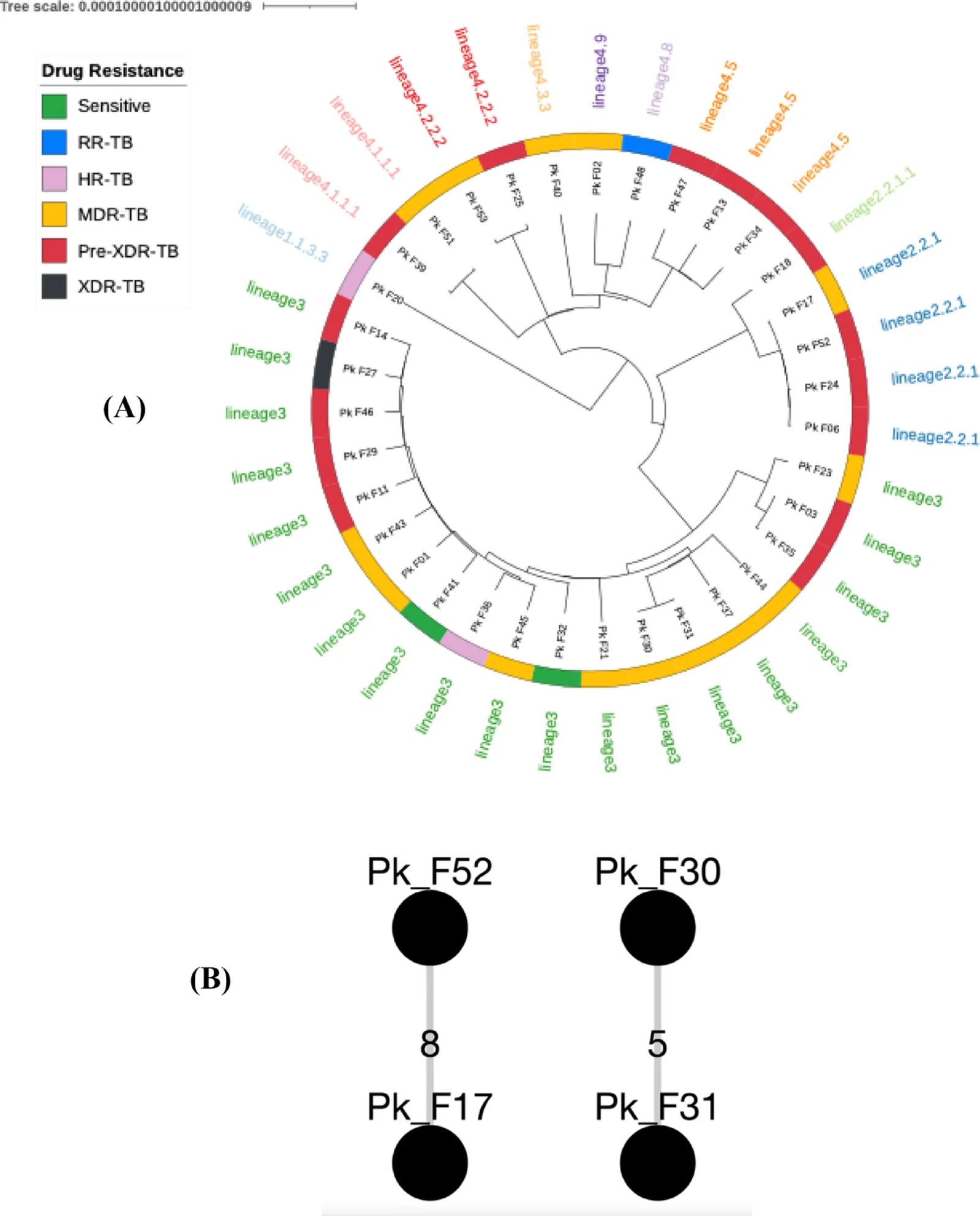
Population structure. (**A**) Maximum-likelihood phylogenetic tree of *M. tuberculosis* isolates (n = 35). (**B**) Potential transmission clusters with isolates within 10 SNP differences.

All thirty-five sequenced isolates exhibited high mapping efficiency, with 97.96%–99.31% of reads aligning to the reference genome and coverage depths ranging from 94- to 428-fold (Supplementary Table 1). The dataset contained 6,635 identified SNPs. In silico lineage analysis showed representation of the four major *M. tuberculosis* lineages, with lineage L3 being the most frequent (n (%): L3, 19 [54.3%]; L4, 10 [28.6%]; L2, 5 [14.3%]; L1, 1 [2.9%]) (Table 1). Most L3 isolates belonged to the CAS1-Delhi strain type (18/19), while all L2 isolates were identified as Beijing strains (L2.2.1). Among L4 isolates, notable diversity was observed, comprising sub-lineages 4.1.1.1, 4.2.2.2, 4.3.3, 4.5, 4.8, and 4.9 (Supplementary Table 1) (Fig. 1).

Analysis of drug resistance mutations revealed 15 pre-XDR-TB (42.9%) and 14 MDR-TB (40.0%) isolates.Additionally, two isolates were isoniazid-resistant TB (HR-TB) and two were pan-sensitive, and one isolate each was categorised as rifampicin-resistant TB (RR-TB) and XDR-TB (Fig. 1).

### Genotypic resistance

Genotypic resistance to isoniazid was detected in 32 isolates, primarily associated with *katG* mutations (S315T in 27/32; T251M in 1/32) and *inhA* promoter variants (− 777C > T in 4/32; − 154G > A in 1/32; − 100C > A in 1/32). Three isolates (Pk_F32, Pk_F41, and Pk_F48) showed discordance between phenotypic and genotypic resistance for isoniazid, potentially indicating phenotypic testing errors. However, Pk_F41 contained an *inhA* promoter mutation (-100C > A); and Pk_F48 was an RR-TB isolate with additional second-line resistance, consistent with previously reported patterns^15^. Rifampicin resistance was identified in 31 isolates (88.6%), predominantly due to mutations in *rpoB*, most commonly S450L (24/31), followed by H445R/Y (3/31), D435V/Y/F (3/31), and S428R (1/31) (Table 2). These mutations are within the 81-bp rifampicin resistance-determining region, corresponding to codons 426–452 in *M. tuberculosis* and corresponding to codons 507–533 in *E. coli*. Ethambutol resistance was predicted in 27 isolates (77.1%), largely driven by *embB* mutations, including M306I/V/L (19/27) and G406A/D (5/27). Pyrazinamide resistance was detected in 20 isolates and was characterised by diverse *pncA* mutations, each observed in at most two isolates.

**Table 2:** Drug resistance mutations underlying genotypic profiles with global frequencies*

Drug	Gene	Change	N	%	Global N*	Global %*
Rifampicin	*rpoB*	S450L	24	68.57	30,940	62.56
Rifampicin	*rpoB*	H445R	2	5.71	453	0.92
Rifampicin	*rpoB*	D435V	1	2.86	3480	7.04
Rifampicin	*rpoB*	D435Y	1	2.86	1163	2.35
Rifampicin	*rpoB*	D435F	1	2.86	159	0.32
Rifampicin	*rpoB*	S450W	1	2.86	559	1.13
Rifampicin	*rpoB*	H445Y	1	2.86	1778	3.59
Rifampicin	*rpoB*	S428R	1	2.86	18	0.04
Isoniazid	ahpC**	− 52C > T	1	2.86	414	0.61
Isoniazid	ahpC**	− 54C > T	1	2.86	125	0.18
Isoniazid	*inhA*	− 777C > T	4	11.43	13,411	19.62
Isoniazid	*inhA*	− 154G > A	1	2.86	1785	2.61
Isoniazid	*katG*	S315T	26	74.29	42,411	62.03
Isoniazid	*katG*	T251M	1	2.86	2	< 0.01
Ethambutol	*embB*	M306I	10	28.57	8849	21.64
Ethambutol	*embB*	M306V	8	22.86	12,210	29.87
Ethambutol	*embB*	G406A	4	11.43	1805	4.42
Ethambutol	*embB*	Q497R	2	5.71	2928	7.16
Ethambutol	*embB*	D354A	2	5.71	1295	3.17
Ethambutol	*embB*	M306L	1	2.86	637	1.56
Ethambutol	*embB*	G406D	1	2.86	1301	3.18
Ethambutol	*embB*	Q497K	1	2.86	607	1.48
Streptomycin	*gid*	102delG	2	5.71	1503	3.31
Streptomycin	*gid*	A200E	1	2.86	43	0.09
Streptomycin	*gid*	351delG	1	2.86	838	1.84
Streptomycin	*gid*	115delC	1	2.86	913	2.01
Streptomycin	*rpsL*	K43R	4	11.43	21,486	47.26
Streptomycin	*rpsL*	K88R	2	5.71	5113	11.25
Streptomycin	*rrs*	514A > C	7	20	4394	9.67
Streptomycin	*rrs*	517C > T	2	5.71	1923	4.23
Streptomycin	*rrs*	906A > G	1	2.86	294	0.65
Fluoroquinolones	*gyrA*	D94G	8	22.86	6096	37.08
Fluoroquinolones	*gyrA*	A90V	5	14.29	3991	24.28
Fluoroquinolones	*gyrA*	D94N	3	8.57	1179	7.17
Fluoroquinolones	*gyrA*	D94A	2	5.71	1571	9.56
Fluoroquinolones	*gyrA*	S91P	1	2.86	928	5.64
Fluoroquinolones	*gyrA*	D94Y	1	2.86	691	4.20
Fluoroquinolones	*gyrA*	G88C	1	2.86	157	0.95
Pyrazinamide	*pncA*	349dupC	2	5.71	5	0.01
Pyrazinamide	*pncA*	Y34S	2	5.71	9	0.03
Pyrazinamide	*pncA*	V180F	2	5.71	101	0.30
Pyrazinamide	*pncA*	D12A	1	2.86	231	0.68
Pyrazinamide	*pncA*	391dupG	1	2.86	147	0.43
Pyrazinamide	*pncA*	D8Y	1	2.86	9	0.03
Pyrazinamide	*pncA*	C14G	1	2.86	107	0.31
Pyrazinamide	*pncA*	H71R	1	2.86	225	0.66
Pyrazinamide	*pncA*	P54L	1	2.86	152	0.45
Pyrazinamide	*pncA*	C14Y	1	2.86	45	0.13
Pyrazinamide	*pncA*	G78S	1	2.86	27	0.08
Pyrazinamide	*pncA*	V131F	1	2.86	18	0.05
Pyrazinamide	*pncA*	Y103C	1	2.86	48	0.14
Pyrazinamide	*pncA*	Y34D	1	2.86	141	0.41
Pyrazinamide	*pncA*	T160P	1	2.86	109	0.32
Pyrazinamide	*pncA*	T47P	1	2.86	50	0.15
Ethionamide	*ethA*	703delT	2	5.71	133	0.37
Ethionamide	*ethA*	Q269*	1	2.86	38	0.11
Ethionamide	*ethA*	754_755dupGC	1	2.86	56	0.16
Ethionamide	*inhA*	− 777C > T	4	11.43	13,411	37.76
Ethionamide	*inhA*	− 154G > A	1	2.86	1785	5.03
Bedaquiline	*mmpR5*	140dupA	1	2.86	0	0.00
Bedaquiline	*mmpR5*	138_139dupTG	1	2.86	15	1.09
Clofazimine	*mmpR5*	138_139dupTG	1	2.86	15	1.12
Clofazimine	*mmpR5*	140dupA	1	2.86	0	0.00
Kanamycin	*eis*	− 10G > A	1	2.86	1207	9.48

* from a global TB dataset (n = 152,408), ** likely compensatory mutations.

Streptomycin resistance was observed in 20 isolates (57.1%) and associated with mutations in *gid* (e.g., 102delG, 2/20), *rpsL* (K43R, 4/20; K88R, 2/20), and *rrs* (514A > C, 7/20; 517C > T, 2/20) (Table 2). Fluoroquinolone resistance occurred in 18 isolates, primarily driven by *gyrA* variants (D94G, 8/18; A90V, 5/18; D94N/Y/A, 7/18), overlapping with the six moxifloxacin-resistant phenotypes. Mutations in *mmpR5* associated with bedaquiline and clofazimine cross-resistance (138_139dupTG, 140dupA) were found in the single isolate predicted to be XDR-TB. Kanamycin resistance was predicted in one isolate via an *eis* -10G > A promoter mutation; no known amikacin resistance markers were detected. Ethionamide resistance was predicted in eight isolates, driven by a combination of *inhA* promoter variants (− 777C > T, − 154G > A) and *ethA* mutations (703delT, Q269*, 754_755dupGC). All variants, except *mmpR5* 140dupA, are represented in a global dataset of 152,408 isolates^26,27^ (Table 2).

### Putative drug resistance mutations

Several mutations classified as having “Uncertain significance” in the WHO catalogue, but which could confer drug resistance, were also analysed for their presence in candidate resistance genes. The frequency of these mutations was assessed in a global dataset of 152,408 *M. tuberculosis* isolates, stratified by drug resistance category (Sensitive, pre-MDR-TB, MDR-TB, pre-XDR-TB, XDR-TB) (Table 3). For isoniazid, three *katG* mutations (A109T, F272L, and G299S) were each detected in a single isolate. Notably, A109T co-occurred with an *ahpC* compensatory mutation (-54C > T). As described above, an additional mutation was identified upstream of *inhA* (− 100C > A), a promoter region targeted by both isoniazid and ethionamide. The frequency of this mutation increased with progression to more advanced forms of drug-resistant TB. For ethambutol, three mutations were identified in *embA*—two in the promoter region (− 11C > T, − 8C > T) and one in the coding region (G759R)—and one in *embB* (F330L). In addition, the *ubiA* mutation A237V was designated a potential resistance determinant due to its enrichment in highly resistant TB isolates. For pyrazinamide, two mutations in *pncA* were identified (C14G and C14Y), both located within codon 14. Several mutations were also detected in the streptomycin resistance gene *gid* (F12S, L44P, G117R, and A205E), collectively occurring across five isolates (Fig. 1).

**Table 3:** Putative resistance mutations with their frequency in a global dataset (n = 152,408).

Gene	Change	N	Sens	Pre-MDR-TB	MDR-TB	Pre-XDR-TB	XDR-TB	Other	WHO*	Drug
*ald*	A183V	1	0.01	0.01	0.04	0.12	0.06	0	N	Cycloserine
*ald*	− 80delA	1	0	0.01	0	0.06	0	0.01	N	Cycloserine
*embA*	G759R	4	0	0.01	0.04	0.07	0	0	Y	Ethambutol
*embA*	− 8C > T	2	0	0.04	0.33	0.46	0.4	0	Y	Ethambutol
*embA*	− 11C > T	1	0	0.03	0.29	0.21	0.51	0	Y	Ethambutol
*embB*	F330L	1	0	0.01	0.07	0.14	0.06	0	Y	Ethambutol
*ethA*	C137R	1	0	0.1	0.18	0.29	0.17	0	Y	Ethionamide
*gid*	F12S	2	0.01	0.01	0.02	0.03	0	0	Y	Streptomycin
*gid*	L44P	1	0.01	0.02	0.02	0.01	0.06	0	Y	Streptomycin
*gid*	G117R	1	0.03	0.11	0.1	0.05	0.06	0.01	Y	Streptomycin
*gid*	A205E	1	0	0	0.02	0.01	0	0	Y	Streptomycin
*inhA*	− 100C > A	1	0.02	0.05	0.1	0.22	0.34	0.01	Y	Isoniazid
*katG*	A109T	1	0.01	0.09	0.11	0.18	0	0	Y	Isoniazid
*katG*	G299S	1	0	0.06	0.01	0.03	0	0	Y	Isoniazid
*katG*	F272L	1	0	0.01	0	0	0.06	0	Y	Isoniazid
*mmpR5*	P14S	1	0	0	0	0.01	0	0	N	Bedaquiline
*pncA*	C14G	1	0	0.02	0.2	0.27	0.4	0	Y	Pyrazinamide
*pncA*	C14Y	1	0	0.01	0.04	0.22	0.06	0	Y	Pyrazinamide
*ribD*	E27D	2	0	0.01	0	0.01	0	0	N	PAS
*rrl*	2295A > C	1	0	0	0	0	0.46	0	Y	Linezolid
*thyA*	− 42C > G	1	0	0	0.01	0.06	0.34	0	N	PAS
*thyA*	A262V	1	0.01	0	0.04	0.05	0.06	0.01	N	PAS
*thyA*	T22N	1	0	0	0.01	0.01	0.23	0	N	PAS
*thyX*	− 9G > A	1	0.01	0.17	0.46	0.9	0.91	0.02	N	PAS
*ubiA*	A237V	1	0	0.02	0.44	0.37	0.17	0	Y	Ethambutol

* present in the WHO mutation list (uncertain); MDR-TB, multidrug-resistant TB; XDR-TB, extensively drug-resistant TB.

**Fig. 2:**
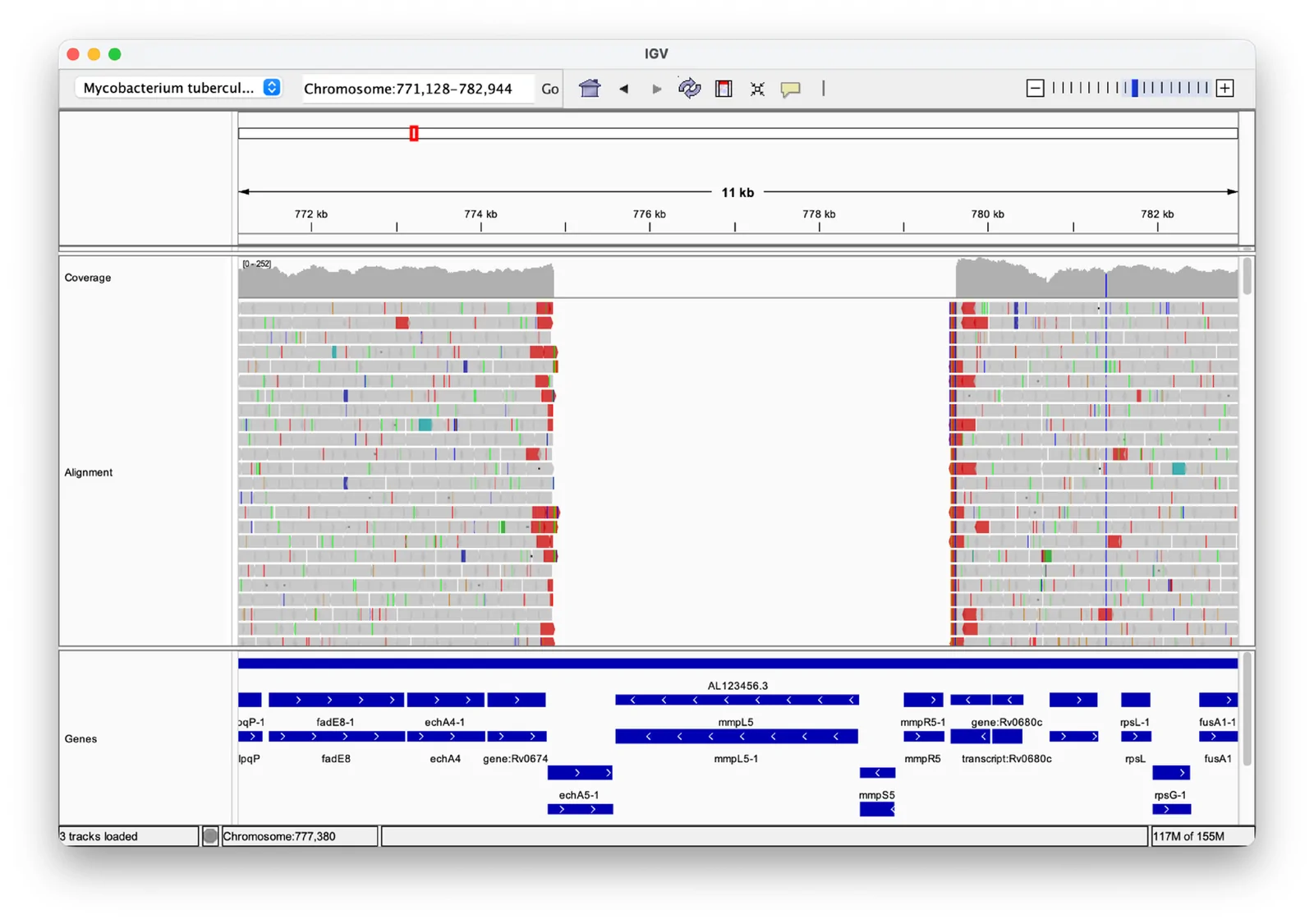
Coverage plot of isolate Pk_F29 with *mmpR5*, *mmpL5* and *mmpS5* deleted, potentially resulting in bedaquiline sensitivity.

For bedaquiline, a SNP in *mmpR5* (P14S) was identified. Although rare in the global dataset, this variant showed slight enrichment among pre-XDR isolates (Table 3). For linezolid, a mutation in *rrl* (2295A > C) was detected and is considered a potential resistance determinant. In the case of ethionamide, a mutation in *ethA* (C137R) was observed, which was enriched in advanced forms of drug-resistant TB in the global dataset. Mutations were also detected in candidate resistance genes for drugs not currently represented in the WHO catalogue. For cycloserine, two mutations were identified in *ald*: one in the promoter region (−80delA) and one in the coding region (A183V). For para-aminosalicylic acid (PAS), mutations were identified in *ribD* (E27D), *thyA* (-42C > G, A262V, T22N), and *thyX* (− 9G > A). Finally, a large deletion was detected in one isolate completely removed *mmpR5* (Fig. 2). While such a deletion would typically confer resistance to bedaquiline and clofazimine, in this case, it also encompassed *mmpL5* and *mmpS5*, likely rendering the isolate bedaquiline-sensitive.

### Phylogenetic tree analysis

A maximum-likelihood phylogenetic tree was constructed using 6,635 SNPs, confirming the expected lineage-based clustering (Fig. 1). The tree topology identifies several closely related isolate clusters, including a tight clade containing isolates Pk_F06, Pk_F17, Pk_F24, and Pk_F52, characterised by extremely short branch lengths. Isolate pairs such as Pk_F30 and Pk_F31, as well as Pk_F52 and Pk_F17, are separated by minimal genetic distances (< 10 SNPs) with similar drug resistance profiles, potentially reflecting direct transmission chains within localised outbreaks (Fig. 1). In contrast, isolate Pk_F20 is highly divergent, as indicated by a long branch.

## Discussion

The epidemiology of drug-resistant TB in Khyber Pakhtunkhwa (KP), Pakistan, reveals a concerning situation with critical implications for clinical and public health sectors. Multidrug-resistant TB (MDR-TB) prevalence is high in KP, exceeding the national average^14^, and the region is known as a hotspot for drug-resistant TB transmission^2,15^. In our study, cases were from both urban (Peshawar) and rural (Dera Ismail Khan, Swat) districts, indicating potential dissemination of resistant strains across diverse geographic and socioeconomic contexts. A limitation of our study is the use of convenience sampling for isolate collection, which prevents us from reliably estimating the prevalence of drug resistance or the distribution of underlying resistance-associated mutations.

The geographic distribution of MDR-TB cases reveals important transmission patterns. Peshawar, the most populous urban centre, accounted for the highest absolute number of cases (n = 9), consistent with typical TB epidemiology where crowded living conditions facilitate transmission^3^. The presence of MDR-TB in all seven surveyed districts, including relatively remote areas like Karak and Bannu, indicates that drug resistance is not confined to urban centres but is present across KP. This widespread distribution aligns with recent reports of expanding drug-resistant TB in Pakistan’s periphery^13^, potentially reflecting population mobility and inconsistent treatment adherence in rural areas. Notably, pre-XDR-TB and XDR-TB cases were identified. The presence of fluoroquinolone resistance is consistent with previous studies and likely reflects the impact of unregulated antibiotic use in KP^31^. One isolate from Peshawar, resistant to both fluoroquinolones and kanamycin, represents a serious therapeutic challenge, as these drugs constitute the backbone of second-line regimens^15^. Larger-scale studies in KP are essential to develop a more comprehensive understanding of the transmission dynamics of drug-resistant TB.

The WGS analysis reveals a complex molecular epidemiology of drug-resistant tuberculosis in KP, characterised by both canonical resistance mutations and emerging patterns. The predominance of *rpoB* S450L (24/31) as the primary rifampicin resistance mechanism aligns with global reports^5^, and previous studies in Pakistan^13,15^. The identified *rpoB* mutations associated with high-level resistance suggest that most cases in this cohort would fail standard rifampicin-containing regimens^32^. Regarding isoniazid resistance, the dual mechanisms of *katG* S315T and *inhA* promoter mutations have important diagnostic implications. While *katG* mutations confer high-level resistance, *inhA* mutations cause low-level resistance and cross-resistance to ethionamide^33^.These findings suggest that some isolates may respond to high-dose isoniazid but not to ethionamide therapy, complicating MDR-TB treatment strategies.

Pyrazinamide resistance exhibited substantial genetic diversity, with > 15 distinct *pncA* mutations distributed across the entire gene, and a maximum frequency of 2. This heterogeneity exceeds that reported in other high-burden regions and complicates molecular detection strategies^34^. The lack of a dominant mutation suggests multiple independent resistance emergence events rather than clonal spread. Fluoroquinolone resistance patterns present significant clinical concerns. The *gyrA* D94G/N/A/Y mutations confer high-level resistance to all fluoroquinolones, whereas A90V confers intermediate resistance^30,35^. These findings suggest that pre-XDR cases in this cohort would not respond to moxifloxacin-based regimens. Shared *mmpR5* mutations in bedaquiline/clofazimine-resistant isolates (c.138_139dupTG, c.140dupA) represent a critical therapeutic challenge^36^. Interestingly, one isolate carried a previously unreported deletion spanning *mmpR5*, *mmpL5*, and *mmpS5*, which may increase susceptibility to bedaquiline; however, this observation requires confirmation through functional studies.

Several putative novel resistance determinants were identified based on their classification as “Uncertain significance” in the WHO catalogue (2nd edition)^37^ and their association with advanced forms of drug-resistant TB (MDR-TB, pre-XDR-TB, and XDR-TB) in a curated global dataset comprising over 152,000 clinical isolates with WGS data^26,27^. Three *katG* mutations (A109T, F272L, and G299S) were detected each contributing to isoniazid resistance. Although most isoniazid resistance is attributed to the canonical *katG* S315T mutation, rare variants can also confer resistance, likely through KatG loss-of-function changes that impose a higher fitness cost^38^. Supporting this interpretation, the isolate carrying *katG* A109T also harboured the known compensatory mutation *ahpC* -54C > T. Another potential resistance mechanism was observed upstream of *inhA* (-100C > A). This variant causes a synonymous substitution in *fabG1* but may increase *inhA* expression by creating an alternative promoter near codon 203 of *fabG1*, approximately 154 nucleotides upstream of *inhA*^39^. The *inhA* -100C > A mutation was enriched in drug-resistant isolates, reaching a frequency of 0.33% among XDR-TB strains in the global dataset^26,27^. Its presence across multiple sublineages suggests independent acquisition events consistent with positive selection. The functional consequences of these mutations on protein structures have been extensively explored through in silico drug docking studies, including several focused on *M. tuberculosis* in Pakistan^16–18^.

A mutation in *rrl* (2295A > C) wasidentified and may confer linezolid resistance. Several *rrl* mutations previously linked to linezolid resistance^37^ span positions 2270 to 2814 of the gene. Although rare, the 2295A > C mutation was observed exclusively in XDR-TB isolates from four distinct sublineages^26,27^, suggesting its potential as a strong candidate for further functional validation. Additional mutations were identified in *pncA* and *gid*, genes where loss-of-function variants confer resistance to pyrazinamide and streptomycin, respectively. Since such variants can occur throughout these genes, the identification of novel, rare mutations is anticipated. Finally, mutations were detected in *thyA*, *thyX*, and *ribD*, genes implicated in PAS resistance. As the PAS drug is not currently included in the 2nd edition of the WHO catalogue^37^, these mutations are reported here as novel candidates pending systematic functional and statistical evaluation.

A limitation of the convenience drug-resistant sampling frame is the potential underrepresentation of lineages less prone to drug resistance. Lineage L3 (CAS/Delhi) isolates dominated our convenience sample, which is consistent with known South Asian TB epidemiology^9^. In contrast, L2 (Beijing), a hypervirulent, drug-resistant strain, was detected at higher frequencies than reported in earlier spoligotyping-based reports^40^. This may reflect differences in sampling, expanded transmission, or improved detection through WGS^27^. The presence of multiple sub-lineages, particularly within L4, suggests complex transmission dynamics, likely reflecting either multiple introductions or long-term endemic circulation in the region. The detection of Beijing lineage strains, all classified as MDR or pre-XDR, is particularly concerning. These strains are characterized by their hyper-transmissibility, increased potential for developing drug resistance, and an association with younger patient populations^7,41^. Their presence suggests the potential for more aggressive outbreaks in the region, warranting heightened public health attention. Phylogenetic analysis identified clusters of closely related isolates (e.g., Pk_F30 and Pk_F31, Pk_F52 and Pk_F17), differing by less than 10 SNPs, indicative of recent person-to-person transmission^7^. These clustered isolates, originating from adjacent districts and sharing identical MDR profiles, including *gyrA* mutations, likely represent localised transmission events rather than independent acquisition of resistance. The Pk_F30 and Pk_F31 isolates may reflect direct transmission or a common exposure source. Conversely, the highly divergent Pk_F20 strain, characterised by a long phylogenetic branch, may represent reactivation of latent infection, an imported case from another region, or an isolate following a unique evolutionary path potentially shaped by host adaptation^42^. These findings underscore the importance of integrating genomic surveillance with detailed patient histories and regional movement data.

Resistance profiles exhibited lineage-specific trends. Beijing strains showed uniformly high levels of resistance, consistent with previous reports linking this lineage to MDR and pre-XDR phenotypes^7^. CAS lineage isolates showed higher ethambutol resistance (83%) than Euro-American (Lineage L4) strains, whereas L4 isolates displayed more heterogeneous resistance patterns and a lower frequency of XDR phenotypes. These differences suggest that lineage background may influence both drug resistance development and transmission potential, with significant implications for treatment strategy and surveillance priorities. These findings highlight several public health needs. Targeted interventions in Beijing lineage hotspots could mitigate the risk of high-transmission, high-resistance outbreaks. Enhanced contact tracing, especially for clustered cases, may allow earlier interruption of transmission chains. The identification of divergent strains highlights the need to assess travel history and possibly implement cross-border screening. Additionally, laboratory protocols should be reviewed to minimise the risk of cross-contamination in genomic workflows, and to ensure robust pDST characterisation. Unlike isoniazid and rifampicin, phenotypic drug resistance to pyrazinamide and ethambutol appears to be underestimated relative to genotypic profiling, reflecting a known limitation of current laboratory assays.

This study has several limitations, including the absence of patient contact data to confirm transmission events, potential sampling bias towards treatment-failure cases, and the lack of environmental or household sampling. Future research should expand sampling to include close contacts, integrate spatial and temporal metadata into genomic analyses, investigate host genetic factors influencing TB outcomes, and assess the in vivo fitness of the dominant sub-lineages circulating in KP. Despite the lack of larger-scale studies, our findings highlight the persistent challenge of drug-resistant TB in the region and demonstrate how WGS can clarify circulating resistance mutations, inform effective therapeutic options, and identify transmission hotspots to guide targeted interventions.

## Supplementary Information

Below is the link to the electronic supplementary material.

Supplementary Material 1

## Data Availability

ENA study accession numbers for the newly generated datasets are provided in Supplementary Table 1.
