# Supplementary material for: Whole-genome analysis of drug resistance and transmission patterns in Mycobacterium tuberculosis from Khyber Pakhtunkhwa, Pakistan
Source: Sci Rep. 2026 Jul 22;16:33296. doi: 10.1038/s41598-026-62482-7 (PMC13686930; doi:10.1038/s41598-026-62482-7)
Supplement: Supplementary file 1 [file 41598_2026_62482_MOESM1_ESM.pdf]

Supplementary Table 1
ENA Accession identifiers

Sample ID	Sample	Seq. depth	% mapped	Lineage	Spoligotype	Family	Drug resist.
Pk_F01	ERS26976205	277	99.13	L3	703600340003771	-	MDR-TB
Pk_F02	ERS26976197	201	99.31	L4.9	477777777760771	T1	MDR-TB
Pk_F03	ERS26976195	155	99.11	L3	703777740003771	CAS1-Delhi	Pre-XDR-TB
Pk_F06	ERS26976213	245	99.17	L2.2.1	000000000003771	Beijing	Pre-XDR-TB
Pk_F11	ERS26976209	163	98.92	L3	703777740003771	CAS1-Delhi	Pre-XDR-TB
Pk_F13	ERS26976212	184	99.16	L4.5	577777777420771	Ural-2	Pre-XDR-TB
Pk_F14	ERS26976201	283	99.12	L3	703777740003771	CAS1-Delhi	Pre-XDR-TB
Pk_F17	ERS26976189	232	99.12	L2.2.1	000000000003771	Beijing	MDR-TB
Pk_F18	ERS26976204	131	99.09	L2.2.1.1	000000000003771	Beijing	Pre-XDR-TB
Pk_F20	ERS26976198	249	98.83	L1.1.3.3	777776757413771	EAI6-BGD1	HR-TB
Pk_F21	ERS26976215	199	99.18	L3	703777740003771	CAS1-Delhi	MDR-TB
Pk_F23	ERS26976188	202	99.23	L3	703777740003771	CAS1-Delhi	MDR-TB
Pk_F24	ERS26976203	226	99.19	L2.2.1	000000000003771	Beijing	Pre-XDR-TB
Pk_F25	ERS26976208	264	99.05	L4.2.2.2	777777777760771	T1	Pre-XDR-TB
Pk_F27	ERS26976186	196	99.12	L3	703777740003771	CAS1-Delhi	XDR-TB
Pk_F29	ERS26976219	178	98.97	L3	703777740003771	CAS1-Delhi	Pre-XDR-TB
Pk_F30	ERS26976191	197	98.38	L3	703777740003771	CAS1-Delhi	MDR-TB
Pk_F31	ERS26976217	144	99.02	L3	703777740003771	CAS1-Delhi	MDR-TB
Pk_F32	ERS26976207	214	99.18	L3	703777740003771	CAS1-Delhi	Sensitive
Pk_F34	ERS26976206	240	99.21	L4.5	577777777420771	Ural-2	Pre-XDR-TB
Pk_F35	ERS26976218	160	99.11	L3	703777740003771	CAS1-Delhi	Pre-XDR-TB
Pk_F36	ERS26976192	228	99.31	L3	703777740003771	CAS1-Delhi	HR-TB
Pk_F37	ERS26976220	152	98.86	L3	703777740003771	CAS1-Delhi	MDR-TB
Pk_F39	ERS26976210	155	99.01	L4.1.1.1	777776777760601	X2	Pre-XDR-TB
Pk_F40	ERS26976190	166	99.15	L4.3.3	777777600000771	LAM	MDR-TB
Pk_F41	ERS26976187	215	99.29	L3	703775740000171	CAS1-Delhi	Sensitive
Pk_F43	ERS26976199	428	98.92	L3	703777700003771	CAS1-Delhi	MDR-TB
Pk_F44	ERS26976211	156	98.28	L3	703777740003771	CAS1-Delhi	MDR-TB
Pk_F45	ERS26976214	94	98.62	L3	703777740003611	CAS1-Delhi	MDR-TB
Pk_F46	ERS26976202	168	98.82	L3	703777740003771	CAS1-Delhi	Pre-XDR-TB
Pk_F47	ERS26976216	236	99.19	L4.5	540017377420771	-	Pre-XDR-TB
Pk_F48	ERS26976193	232	99.19	L4.8	777777777760771	T1	RR-TB
Pk_F51	ERS26976196	193	99.07	L4.1.1.1	777776777760601	X2	MDR-TB
Pk_F52	ERS26976200	157	98.93	L2.2.1	000000000003771	Beijing	Pre-XDR-TB
Pk_F53	ERS26976194	119	97.96	L4.2.2.2	777777777760771	T1	MDR-TB

DR, drug resistance; RR-TB, rifampicin-resistant TB; HR-TB, isoniazid-resistant TB; MDR-TB, multidrug-resistant TB; XDR-TB, extensively drug-resistant TB

Supplementary Table 2

For each isolate, established drug resistance (DR) mutations

Sample ID	DR	No. DR Variants	Mutations
Pk_F01	MDR-TB	5	*rpoB* S450L, *katG* S315T, *pncA* V180F, *embB* M306I, *gid* 115delC
Pk_F02	MDR-TB	5	*rpoB* S450L, *rpsL* K43R, *katG* S315T, *pncA* P54L, *embB* M306V
Pk_F03	Pre-XDR	7	*gyrA* D94G, *rpoB* S450L, *rpsL* K43R, *katG* S315T, *pncA* 349dupC, *embB* M306I, *ethA* 703delT
Pk_F06	Pre-XDR	7	*gyrA* D94G, *rpoB* S450L, *rpoC* I491T, *rrs* 514A>C, *katG* S315T, *pncA* T47P, *embB* M306V
Pk_F11	Pre-XDR	6	*gyrA* A90V, *rpoB* S450L, *rrs* 514A>C, *katG* S315T, *pncA* V131F, *embB* Q497R
Pk_F13	Pre-XDR	9	*gyrA* A90V, *gyrA* D94N, *gyrA* D94G, *rpoB* S450L, *katG* S315T, *pncA* H71R, *embB* M306I, *embB* G406A, *gid* 102delG
Pk_F14	Pre-XDR	5	*gyrA* A90V, *rpoB* S450L, *rrs* 906A>G, *katG* S315T, *embB* G406A
Pk_F17	MDR-TB	6	*rpoB* S450L, *rpoC* I491T, *rrs* 514A>C, *katG* S315T, *pncA* T160P, *embB* M306V
Pk_F18	Pre-XDR	4	*gyrA* G88C, *rpoB* S450L, *katG* S315T, *embB* G406A, *pncA* 119_130delACTACCATCACG
Pk_F20	HR-TB	3	*gyrA* A90V, *inhA* -777C>T, *katG* T251M
Pk_F21	MDR-TB	3	*rpoB* D435Y, *rrs* 517C>T, *katG* S315T
Pk_F23	MDR-TB	4	*rpoB* S450L, *katG* S315T, *pncA* D8Y, *embB* D354A
Pk_F24	Pre-XDR	7	*gyrA* D94G, *rpoB* S450L, *rpoC* I491T, *rrs* 514A>C, *katG* S315T, *pncA* V180F, *embB* M306V
Pk_F25	Pre-XDR	5	*gyrA* D94A, *rpoB* D435V, *katG* S315T, *pncA* Y103C, *embB* M306I
Pk_F27	XDR-TB	5	*gyrA* A90V, *rpoB* S450L, *mmpR5* 138_139dupTG, mmpR5 140dupA, *inhA* -777C>T
Pk_F29	Pre-XDR	6	*gyrA* D94Y, *rpoB* S428R, *rpoB* H445Y, *embB* Q497R, *katG* T376S, *katG* G299S
Pk_F30	MDR-TB	3	*rpoB* H445R, *katG* S315T, *embB* M306V
Pk_F31	MDR-TB	3	*rpoB* H445R, *katG* S315T, *embB* M306V
Pk_F32	Sensitive	0	-
Pk_F34	Pre-XDR	10	*gyrA* D94N, *rpoB* S450L, *rrs* 514A>C, *inhA* -777C>T, *pncA* G78S, *ahpC* -54C>T, *embB* M306I, *embB* G406D, *ethA* Q269*, *gid* A200E
Pk_F35	Pre-XDR	7	*gyrA* D94G, *rpoB* S450L, *rpsL* K43R, *katG* S315T, *pncA* 349dupC, *embB* M306I, *ethA* 703delT
Pk_F36	HR-TB	2	*gyrA* D94G, *inhA* -777C>T
Pk_F37	MDR-TB	4	*rpoB* S450W, *rpsL* K43R, *katG* S315T, *embB* G406A
Pk_F39	Pre-XDR	5	*gyrA* D94G, *rpoB* S450L, *katG* S315T, *pncA* Y34S, *embB* M306I
Pk_F40	MDR-TB	5	*rpoB* S450L, *inhA* -154G>A, *katG* S315T, *pncA* Y34D, *embB* M306V
Pk_F41	Sensitive	0	-
Pk_F43	MDR-TB	5	*rpoB* S450L, *rrs* 517C>T, *katG* S315T, *pncA* D12A, *embB* M306L
Pk_F44	MDR-TB	5	*rpoB* D435F, *rrs* 514A>C, *katG* S315T, *pncA* C14G, *embB* M306I
Pk_F45	MDR-TB	4	*rpoB* S450L, *rpoC* D747A, *katG* S315T, *gid* 351delG
Pk_F46	Pre-XDR	5	*gyrA* D94G, *rpoB* S450L, *rpsL* K88R, *katG* S315T, *embB* D354A
Pk_F47	Pre-XDR	5	*gyrA* D94A, *rpoB* S450L, *pncA* 391dupG, *ahpC* -52C>T, *embB* M306I
Pk_F48	RR-TB	4	*rpoB* S450L, *rpsL* K88R, *eis* -10G>A, *ethA* 754_755dupGC
Pk_F51	MDR-TB	4	*rpoB* S450L, *katG* S315T, *pncA* Y34S, *embB* M306I
Pk_F52	Pre-XDR	8	*gyrA* S91P, *gyrA* D94N, *rpoB* S450L, *rpoC* I491T, *rrs* 514A>C, *katG* S315T, *pncA* C14Y, *embB* M306V
Pk_F53	MDR-TB	4	*rpoB* S450L, *katG* S315T, *embB* Q497K, *gid* 102delG

RR-TB, rifampicin-resistant; HR-TB, isoniazid-resistant; MDR-TB, multidrug-resistant; XDR-TB, extensively drug-resistant.

Supplementary Table 3
Mutations identified in candidate drug resistance loci, with their frequencies in the global dataset*

Gene	Change	N	WHO	Sens.	Pre-MDR-TB	MDR-TB	Pre-XDR-TB	XDR-TB	Other
katG	S315T	26	Y	0	39.24	81.30	84.03	83.92	0
rpoB	S450L	24	Y	0	7.81	64.17	71.93	60.03	0
embB	M306I	10	Y	0	2.28	16.59	21.84	32.10	0.3
embB	M306V	8	Y	0	2.02	22.23	34.56	28.51	0.25
gyrA	D94G	8	Y	0	0.55	0.02	34.04	40.65	1.98
rrs	514A>C	7	Y	0	2.14	5.66	12.14	22.46	2.05
gyrA	A90V	5	Y	0	0.42	0	23.35	17.16	1.32
embA	G759R	4	Y	0	0.01	0.04	0.07	0	0
embB	G406A	4	Y	0	0.31	4.27	3.30	3.19	0.06
inhA	-777C>T	4	Y	0	22.28	20.41	27.95	21.78	0
rpsL	K43R	4	Y	0	13.74	35.78	47.75	39.79	11.14
fbiB	K448R	3	Y	0.71	0.56	0.43	0.65	1.37	0.46
gyrA	D94N	3	Y	0	0.11	0	6.44	8.44	0.46
Rv1258c	P414S	3	Y	0.7	0.48	0.08	0.29	0.06	0.42
embA	-8C>T	2	Y	0	0.04	0.33	0.46	0.40	0
embB	Q497R	2	Y	0	0.61	4.63	9.24	8.89	0.09
embB	D354A	2	Y	0	0.31	3.06	2.40	1.54	0.03
fbiA	T302M	2	Y	0.98	1.5	0.61	0.36	0.34	0.49
gid	R83G	2	Y	0.04	0.06	0.05	0.04	0	0
gid	F12S	2	Y	0.01	0.01	0.02	0.03	0	0
gyrA	D94A	2	Y	0	0.13	0	9.16	9.35	0.29
mshA	A134V	2	Y	0.01	0	0	0	0.23	0.01
pncA	V180F	2	Y	0	0.04	0.2	0.26	0	0
pncA	Y34S	2	Y	0	0	0.03	0.01	0	0
rpoB	H445R	2	Y	0	0.28	1.06	0.70	0.46	0
rpsL	K88R	2	Y	0	4.14	9.66	9.29	6.33	2.08
rrs	517C>T	2	Y	0	0.76	3.77	3.79	4.33	0.75
ahpC	-52C>T	1	Y	0	0.5	0.88	0.60	0.29	0
ahpC	-54C>T	1	Y	0	0.19	0.2	0.26	0.11	0
clpC1	E793K	1	Y	0.01	0	0	0.01	0	0
clpC1	T208I	1	Y	0.01	0	0	0.01	0	0.01
eis	-10G>A	1	Y	0	0.19	1.98	3.73	3.88	0.03
eis	P193S	1	Y	0.04	0	0.01	0.01	0	0.03
embA	N629S	1	Y	0	0	0	0.03	0	0
embA	-11C>T	1	Y	0	0.03	0.29	0.21	0.51	0
embA	A409T	1	Y	0.01	0	0.01	0.01	0	0.01
embA	Q57E	1	Y	0	0	0	0.02	0	0
embB	M306L	1	Y	0	0.18	1.26	1.50	1.60	0.03

embB	F330L	1	Y	0	0.01	0.07	0.14	0.06	0
embB	T546A	1	Y	0.01	0	0	0	0	0
embB	Q497K	1	Y	0	0.19	0.96	1.42	4.28	0.17
embB	G406D	1	Y	0	0.74	2.88	2.36	1.31	0.06
embC	-467C>A	1	Y	0.11	0.09	0.01	0.01	0	0.11
ethA	C137R	1	Y	0	0.10	0.18	0.29	0.17	0
ethA	Q269*	1	Y	0	0	0.10	0.07	0.06	0
fgd1	T255A	1	Y	0.04	0.04	0	0.01	0	0.03
gid	A205E	1	Y	0	0	0.02	0.01	0	0
gid	L44P	1	Y	0.01	0.02	0.02	0.01	0.06	0
gid	D85A	1	Y	0.01	0.01	0.02	0.01	0	0.01
gid	T146P	1	Y	0	0	0.01	0	0	0.01
gid	L145S	1	Y	0.01	0	0.06	0	0	0.01
gid	V88A	1	Y	0.01	0.04	0.05	0	0	0
gid	G117R	1	Y	0.03	0.11	0.10	0.05	0.06	0.01
gid	L142S	1	Y	0	0	0.01	0	0	0
gid	A200E	1	Y	0	0.01	0.07	0.06	0	0.08
gid	S70I	1	Y	0.01	0.06	0.01	0	0	0
gyrA	S91P	1	Y	0	0.05	0	5.15	5.87	0.4
gyrA	L346V	1	Y	0.05	0.01	0.01	0.04	0	0.01
gyrA	G88C	1	Y	0	0	0	0.91	0.63	0.09
gyrA	D94Y	1	Y	0	0.14	0	3.72	5.07	0.24
gyrB	A398V	1	Y	0.06	0.04	0.01	0	0	0.03
gyrB	S363R	1	Y	0.01	0	0	0.01	0	0
gyrB	S629A	1	Y	0	0	0	0	0	0.01
inhA	-100C>A	1	Y	0.02	0.05	0.1	0.22	0.34	0.01
inhA	-154G>A	1	Y	0	3.41	2.64	3.21	4.85	0.01
katG	T251M	1	Y	0	0	0.01	0	0	0
katG	-482T>G	1	Y	0	0.01	0	0.01	0	0
katG	Q679Y	1	Y	0	0	0	0	0	0
katG	F272L	1	Y	0	0.01	0	0	0.06	0
katG	G299S	1	Y	0	0.06	0.01	0.03	0	0
katG	A109T	1	Y	0.01	0.09	0.11	0.18	0	0
mmpL5	E552G	1	Y	0.11	0.09	0.01	0.01	0	0.11
mshA	-632G>A	1	Y	0.01	0.01	0.01	0.03	0.34	0
pepQ	R7Q	1	Y	0.07	0.06	0.03	0.03	0	0.09
pncA	C14Y	1	Y	0	0.01	0.04	0.22	0.06	0
pncA	H71R	1	Y	0	0.03	0.4	0.68	0.34	0.01
pncA	V131F	1	Y	0	0	0.03	0.03	0.23	0
pncA	D12A	1	Y	0	0.04	0.47	0.54	0.06	0.07
pncA	T47P	1	Y	0	0	0.09	0.17	0	0
pncA	P54L	1	Y	0	0.01	0.28	0.42	0.34	0.01

pncA	T160P	1	Y	0	0.01	0.24	0.20	0.63	0
pncA	Y34D	1	Y	0	0.01	0.37	0.17	0.51	0
pncA	G78S	1	Y	0	0	0.04	0.12	0	0
pncA	D8Y	1	Y	0	0	0.02	0.01	0	0
pncA	C14G	1	Y	0	0.02	0.20	0.27	0.4	0
pncA	Y103C	1	Y	0	0.01	0.08	0.15	0.11	0
rpoB	R552L	1	Y	0	0	0.10	0.12	0.06	0
rpoB	S428R	1	Y	0	0	0.05	0.02	0	0
rpoB	H445Y	1	Y	0	2.4	3.86	2.06	2.51	0
rpoB	D435Y	1	Y	0	0.83	2.48	1.84	4.16	0
rpoB	I488V	1	Y	0	0.01	0.39	0.3	0.17	0
rpoB	S450W	1	Y	0	0.31	1.39	0.77	0.40	0
rpoB	D435F	1	Y	0	0.11	0.36	0.29	0	0
rpoB	D435V	1	Y	0	0.41	7.01	7.86	15.79	0
rrl	2295A>C	1	Y	0	0	0	0	0.46	0
rrl	2262C>A	1	Y	0.01	0.02	0	0	0	0
rrl	1351C>A	1	Y	0.06	0.05	0.01	0.01	0.06	0.05
rrl	2050T>C	1	Y	0.03	0.02	0.02	0.01	0	0.01
rrs	906A>G	1	Y	0	0.12	0.58	0.60	0.86	0.05
Rv1258c	P369T	1	Y	0.02	0.01	0.01	0.05	0.11	0
Rv1979c	A66V	1	Y	0	0	0	0.01	0.06	0.01
ubiA	A237V	1	Y	0	0.02	0.44	0.37	0.17	0
whiB7	P11S	1	Y	0.05	0	0.01	0.02	0	0
rrs	-187C>T	35	N	99.89	99.94	99.94	99.82	99.94	99.89
ald	-32T>C	30	N	82.55	85.16	90.11	94.93	95.27	89.95
panD	-591C>T	19	N	11.1	7.62	4.51	4.95	8.38	7.98
pncA	-125delC	19	N	11.33	7.83	4.5	4.97	8.55	8.01
alr	-125G>A	10	N	0	0	0	0	0	0
mmpS5	-710C>G	5	N	21.71	30.38	45.26	67.3	61.86	19.12
Rv1258c	580_581insC	5	N	20.56	28.6	42.11	57.70	39.45	18.53
mmaA3	441C>T	4	N	0.03	0	0.05	0.07	0	0.01
ald	Q56H	2	N	0.62	0.4	0.22	0.31	0.57	0.38
ald	-78A>C	2	N	2.71	3.38	6.72	4.06	2.05	1.68
alr	765T>C	2	N	0	0	0	0	0	0
ethA	703delT	2	N	0	0.02	0.21	0.48	0.06	0
fbiD	216G>A	2	N	0.02	0.10	0	0.01	0	0
gid	102delG	2	N	0	2.29	1.43	1.72	2.17	3.2
inhA	-834A>G	2	N	0.04	0.02	0.01	0.02	0	0.01
pncA	349dupC	2	N	0	0	0.01	0.01	0	0
ribD	E27D	2	N	0	0.01	0	0.01	0	0
rplC	-590T>C	2	N	3.76	3.87	2.92	3.58	4.10	2.03
rplC	-605T>C	2	N	2.7	3.38	6.77	4.10	2.11	1.68

rpoB	A420V	2	N	0	0	0	0	0	0
Rv1258c	225G>A	2	N	0	0	0	0	0	0
ahpC	-142G>A	1	N	11.45	11.44	4.60	1.29	3.36	7.38
ald	A183V	1	N	0.01	0.01	0.04	0.12	0.06	0
ald	-80delA	1	N	0	0.01	0	0.06	0	0.01
alr	-1C>A	1	N	0	0	0	0	0	0
alr	-198C>A	1	N	0	0	0	0	0	0
alr	-265_-264insC	1	N	0	0	0	0	0	0
clpC1	2013C>T	1	N	0	0	0	0	0	0
ddn	456A>C	1	N	0	0	0	0	0	0
embA	V389M	1	N	0	0	0.02	0	0	0
embA	Q756P	1	N	0	0	0	0	0	0
embB	2532G>A	1	N	0	0	0	0	0	0
ethA	754_755dupGC	1	N	0	0.03	0.08	0.19	0	0.01
ethA	E427V	1	N	0	0	0	0	0	0
ethA	L348R	1	N	0	0	0	0	0	0
fbiA	-169A>G	1	N	0.02	0.04	0.01	0.01	0.29	0.06
fbiA	-37G>T	1	N	0	0	0	0	0	0
fgd1	-224G>A	1	N	0.01	0	0	0.03	0.34	0
gid	351delG	1	N	0	1.18	1.06	0.6	0.57	1.82
gid	115delC	1	N	0	0.81	1.01	0.67	1.25	2.59
gyrA	906T>C	1	N	0	0	0	0	0	0.01
gyrB	F383V	1	N	0	0	0	0	0	0
gyrB	H263R	1	N	0	0.01	0	0	0	0
inhA	51C>T	1	N	0	0	0	0	0	0
kasA	18C>T	1	N	13	15.09	5.35	1.31	3.59	48.61
kasA	G269S	1	N	4.84	5.09	8.94	6.03	4.45	1.83
katG	R187Q	1	N	0	0.01	0	0	0	0
katG	T376S	1	N	0	0	0	0	0	0
mmpR5	P14S	1	N	0	0	0	0.01	0	0
mmpR5	138_139dupTG	1	N	0	0	0	0	0.80	0
mmpR5	140dupA	1	N	0	0	0	0	0	0
mmpR5	-4131_*138del	1	N	0	0	0	0	0	0
mshA	R256Q	1	N	0	0	0	0	0	0
panD	-384C>T	1	N	0	0.01	0	0	0	0
pncA	391dupG	1	N	0	0.01	0.18	0.59	0.57	0
pncA	119_130delACTACCATCACG	1	N	0	0	0	0	0	0
ribD	349C>T	1	N	0.07	0.04	0.01	0.03	0	0.05
rpsL	-259C>T	1	N	0.92	2.07	0.56	0.37	0.40	0.27
rrs	187_196dupGATGCATGTC	1	N	0	0	0	0	0	0
thyA	T22N	1	N	0	0	0.01	0.01	0.23	0

thyA	A262V	1	N	0.01	0	0.04	0.05	0.06	0.01
thyA	T202A	1	N	13.73	11.57	15.95	8.97	7.58	3.20
thyA	-42C>G	1	N	0	0	0.01	0.06	0.34	0
thyX	-9G>A	1	N	0.01	0.17	0.46	0.90	0.91	0.02
tlyA	-141T>G	1	N	0.01	0	0	0.01	0	0.01
whiB7	-193C>T	1	N	0	0	0	0	0	0

WHO refers to presence in their mutation list; RR-TB, rifampicin-resistant TB; HR-TB, isoniazid-resistant TB; MDR-TB, multidrug-resistant TB; XDR-TB, extensively drug-resistant TB; * N=152,408
